# Which Single Intervention Would Do the Most to Improve the Health of Those Living on Less Than $1 Per Day?

**DOI:** 10.1371/journal.pmed.0040303

**Published:** 2007-10-23

**Authors:** Gavin Yamey, on Behalf of the Interviewees

## Abstract

Background to the debate: *PLoS Medicine* is participating in the Council of Science Editors' global theme issue on poverty and human development on October 22, 2007 (http://www.councilscienceeditors.org/globalthemeissue.cfm). Over 200 scientific and medical journals are taking part. For our theme issue, we asked a wide variety of commentators worldwide—including clinicians, medical researchers, health reporters, policy makers, health activists, and development experts—to name the single intervention that they think would improve the health of those living in poverty. We also asked four individuals living in poor, rural agricultural communities in the Santillana district, province of Huanta, Ayacucho, Peru to give us their response to the question, “What do you think would do the most to improve your health and the health of your family?” (The four community members were Severino Rojas Poma, Mercedes Vargas Soto, Julián De La Cruz Chahua, and Martín Rojas Poma). Our October 2007 Editorial discusses this debate further.

## Abstract 2

*PLoS Medicine* put this question to a wide variety of commentators worldwide, including medical researchers, policy makers, health reporters, and members of poor rural communities in Peru.

## Zulfiqar Bhutta, Husein Lalji Dewraj Professor of Paediatrics and Child Health, Aga Khan University, Karachi, Pakistan

It is difficult to imagine that people living below the poverty line would be able to “buy” health interventions without assistance, and so the greatest impact may come from giving cash transfers to the poor, conditional on seeking health interventions such as vaccines and nutritional supplements.

## Jeffrey Sachs, Director of the United Nations Millennium Project and Special Advisor to United Nations Secretary-General Kofi Annan on the Millennium Development Goals, Earth Institute, New York, New York, United States of America

In tropical Africa, a mass distribution of free long-lasting insecticide-treated bed nets to fight malaria accompanied by free access to artemisinin-based combination anti-malaria medicines. In other parts of the world, the situation will be different. I should add that I've spent years objecting to posing the question this way, since at low cost we could achieve major health advances through more comprehensive approaches.

## Paul Farmer, Founding Director, Partners in Health and Presley Professor of Medical Anthropology, Harvard Medical School, Boston, USA

Hire community health workers to serve them. In my experience in the rural reaches of Africa and Haiti, and among the urban poor too, the problem with so many funded health programs is that they never go the extra mile: resources (money, people, plans, services) get hung up in cities and towns. If we train village health workers, and make sure they're compensated, then the resources intended for the world's poorest—from vaccines, to bednets, to prenatal care, and to care for chronic diseases like AIDS and tuberculosis—would reach the intended beneficiaries. Training and paying village health workers also creates jobs among the very poorest.

## Armida Fernandez, Retired Professor of Neonatology and Founder Trustee of the Society for Nutrition, Education and Health Action, Mumbai, India

Taking into consideration the immediate, short term and long term benefits of exclusive breastfeeding and the unbelievable cost effectiveness, proven by evidence based data, I feel that exclusive breastfeeding for the first 6 months is the most important intervention that would save lives in populations living on less than one dollar a day.

## Severino Rojas Poma, 42 Years Old, Buena Vista Community, Ayacucho, Peru

Government help/support with food and medicines, especially tonics and vitamins.

## Davidson Gwatkin, Consultant on Health and Poverty, Washington, D. C., United States of America

The health of the world's poor would be best served by a series of revolutions that bring into power national leaderships that are centrally concerned about the well-being of disadvantaged groups within their borders.

## Mushtaque Chowdhury, Director of the Research and Evaluation Division of Bangladesh Rural Advancement Committee, Dhaka, Bangladesh

Ensure two square meals a day; I believe for the poorest food is the most effective first intervention for health improvement.

## Paul Hunt, United Nations Special Rapporteur on the Right to the Highest Attainable Standard of Health, Human Rights Centre, University of Essex, Essex, United Kingdom

An intervention that, firstly, ensures their active and informed participation in health policy-making impacting upon their lives and, secondly, provides them with effective, transparent and accessible mechanisms of accountability enabling them to scrutinize whether or not those in authority have fulfilled their health responsibilities and promises.

## Mercedes Vargas Soto, 32 Years Old, Ccanobamba Community, Ayacucho, Peru

Improve the house, which is small and untidy.

## Rosebell Kagumire, Reporter (Focus on Health and Human Rights), NTV Uganda, Kampala, Uganda

There's a saying that when you educate a woman you have educated a whole village. This is true: once women are in positions of power they can plan better for their communities—they can better manage their health and that of their children. Health in many poor nations is closely linked with personal income and levels of education. An educated woman will know what her child needs to eat for nutritional purposes and her income level is mostly higher than that of illiterate mothers.

## Jaime Miranda, Civil Association for Health and Human Rights Education, EDHUCASalud, Lima, Peru

In the long run, quality education for children and water and sanitation for everyone.

In the medium term, vaccination.

## Geeta Rao Gupta, President of the International Center for Research on Women, Washington, D. C., United States of America

Invest in empowering women. How? Expand savings and credit for women so they have immediate access to emergency funds to pay for treatment—including the purchase of medicines—of catastrophic illness.

## Hector Garcia, Department of Microbiology, Universidad Peruana Cayetano Heredia, Cysticercosis Unit, Instituto de Ciencias Neurologicas, Lima, Peru

The greatest improvement in health will come from general education (i.e., not specifically health education); there will be an initial lag period (which is why politicians do not like it), but after that it should improve income, living conditions and use of health facilities—and money for its implementation can be made available if all sectors force decision makers to stop purchasing weapons.

## Nicholas J. White, Professor of Tropical Medicine, Mahidol University, Bangkok, Thailand

A highly respected, politically independent, empowered, courageous, motivated, transparent, technologically competent, and properly supported World Health Organization.

## Murugi Murekio, Reporter and Editor (Focus on Health, Poverty Reduction, Sustainable Development, Women and Children), Addis Ababa, Ethiopia

We need to ensure food security. Those living in extreme poverty barely eat and due to this they are more prone to succumb to opportunistic infections that greatly weaken their bodies. For example in Ethiopia, antiretrovirals are free, but mostly women can barely afford a meal a day and so this diminishes their capacity to live healthily with HIV because they have no food.

## Julián De La Cruz Chahua, 34 Years Old, San Luis Community, Ayacucho, Peru

Medicines available in the community and an active community health promoter, because we are far away from the health post.

## Richard Smith, Executive Director, Ovations, UnitedHealth Europe, London, United Kingdom, and Member of the Board of Directors, Public Library of Science

The health of the super poor would be most improved by a successful campaign that led rich countries to accept a substantial and continuing transfer of resources to poor countries in the way that the wealthy accept a substantial transfer of resources within countries through taxation.

## Robert Hecht, Senior Vice President for Public Policy, The International AIDS Vaccine Initiative, New York, New York, United States of America

A vaccine to prevent AIDS. The majority of those infected with HIV are poor, and AIDS undermines their efforts to escape from poverty. An AIDS vaccine would be doubly powerful. Not only would it end the worst pandemic of our time and give a huge boost to the economically disadvantaged—it would also revolutionize the way in which the world goes about paying for and distributing new health technologies to solve some of our toughest global challenges.

## Sanjeev Krishna, Professor of Molecular Parasitology and Medicine, St George's Hospital Medical School, London, United Kingdom

The provision of safe water for drinking, cooking and washing may revolutionise the health of millions who have very little at the moment but are denied this unarguable human right.

## Jose Acuin, Consultant, De La Salle University, Cavite, The Philippines

I think that providing basic education and the enabling conditions under which the poorest can have access to this promises to be the single most effective intervention to help them help themselves sustainably and permanently.

## Calestous Juma, Professor of the Practice of International Development, John F. Kennedy School of Government, Harvard University, Cambridge, Massachusetts, United States of America

Building rural road networks and other supportive infrastructure such as clean water supply would do more for health in developing countries in the short-run than much of the investment in developing new vaccines. Building effective rural transportation networks, for example, would extend the use of existing vaccines and would also make it easier to centralize many of the rural clinics into better health facilities. Co-locating educational and health facilities would make it easier to improve health care and health education in rural areas.

## R. Srinivasa Murthy, Office of the World Health Organization Representative in Sudan, Mental Health, Khartoum, Sudan

Education of women has been consistently shown to have a major impact on a number of health conditions. In addition, it can be the basis for self-care and individual empowerment for health, in a world of information technology.

## Salla Munro, Medical Research Council of South Africa, Health Systems Research Unit, Cape Town, South Africa

The intervention I believe will most improve the health of those living on less than $1 per day is—short of job creation—a nutritional intervention, ensuring people in poorer communities get adequate nutrition to improve their immunity and keep diseases, such as tuberculosis, at bay.

## S. V. Subramanian, Associate Professor, Department of Society, Human Development and Health, Harvard School of Public Health, Cambridge, Massachusetts, United States of America

Providing and ensuring adequate and equitable access to clean water and sanitation is likely to yield the greatest health dividend for the world's poor, with the positive social and environmental externalities associated with this intervention additionally providing health benefits for the non-poor.

## Kelley Lee, Centre on Global Change and Health, London School of Hygiene and Tropical Medicine, London, United Kingdom

A genuine commitment by industrialised countries to fair trade and, in particular, to end the destructive impact of agricultural subsidies on the livelihoods of the poor, would greatly enhance household incomes, food security and thus widespread improvements in the health of the poor.

## Thomas Novotny, Director of International Programs, UCSF School of Medicine, and Education Coordinator for UCSF Global Health Sciences, University of California San Francisco, San Francisco, California, United States of America

I believe that focused aid from developed countries to specific designated partner countries for public health infrastructure under a global governance system of administration, amounting to 1 percent of gross domestic product (GDP) on the part of donors would help.

## Martín Rojas Poma, 42 Years Old, Punkumarccari Community, Ayacucho, Peru

Communication and comprehension with my wife and family, and better coordination with neighbors.

## Kumariah Balasubramaniam, Health Action International Asia Pacific, Colombo, Sri Lanka

Implementation of viable land reforms whereby every family owns adequate arable land, legal protection of domestic agriculture, a sustainable nanocredit system, single payer universal health financing system to provide free basic healthcare to all and compulsory, free primary and secondary education. Individually each of these components will have little impact but in combination they will act synergistically to eradicate poverty.

## Helene Gayle, President and CEO, Care USA, Atlanta, Georgia, United States of America

I don't think there is one medical intervention that is the answer, rather improving basic socioeconomic conditions that will facilitate better housing, education, access to clean water, adequate nutrition, etc. will make the biggest improvements in health. I recently went to a village in a rural Bangladeshi community where improvement in the community economic status allowed the community to install pit latrines, leading to changes in under five mortality because of rapid decline in diarrheal disease. Children were then able to go to school where among other things they learned about hygiene and so the family learned as well and illnesses in the family also went down.

## Solomon Benatar, Professor of Medicine, University of Cape Town, Cape Town, South Africa

Only when (and if) the “haves” develop genuine empathy for the “have-nots,” and come to acknowledge their own long-term interdependence with all other humans, will the global economy be improved to any significant advantage for the desperately poor.

